# Involvement of Sigma-1 Receptors in the Antidepressant-like Effects of Dextromethorphan

**DOI:** 10.1371/journal.pone.0089985

**Published:** 2014-02-28

**Authors:** Linda Nguyen, Matthew J. Robson, Jason R. Healy, Anna L. Scandinaro, Rae R. Matsumoto

**Affiliations:** Department of Basic Pharmaceutical Sciences, and Department of Behavioral Medicine and Psychiatry, West Virginia University, Morgantown, West Virginia, United States of America; University of Medicine & Dentistry of NJ - New Jersey Medical School, United States of America

## Abstract

Dextromethorphan is an antitussive with a high margin of safety that has been hypothesized to display rapid-acting antidepressant activity based on pharmacodynamic similarities to the N-methyl-D-aspartate (NMDA) receptor antagonist ketamine. In addition to binding to NMDA receptors, dextromethorphan binds to sigma-1 (σ_1_) receptors, which are believed to be protein targets for a potential new class of antidepressant medications. The purpose of this study was to determine whether dextromethorphan elicits antidepressant-like effects and the involvement of σ_1_ receptors in mediating its antidepressant-like actions. The antidepressant-like effects of dextromethorphan were assessed in male, Swiss Webster mice using the forced swim test. Next, σ_1_ receptor antagonists (BD1063 and BD1047) were evaluated in conjunction with dextromethorphan to determine the involvement of σ receptors in its antidepressant-like effects. Quinidine, a cytochrome P450 (CYP) 2D6 inhibitor, was also evaluated in conjunction with dextromethorphan to increase the bioavailability of dextromethorphan and reduce exposure to additional metabolites. Finally, saturation binding assays were performed to assess the manner in which dextromethorphan interacts at the σ_1_ receptor. Our results revealed dextromethorphan displays antidepressant-like effects in the forced swim test that can be attenuated by pretreatment with σ_1_ receptor antagonists, with BD1063 causing a shift to the right in the dextromethorphan dose response curve. Concomitant administration of quinidine potentiated the antidepressant-like effects of dextromethorphan. Saturation binding assays revealed that a K_i_ concentration of dextromethorphan reduces both the K_d_ and the B_max_ of [^3^H](+)-pentazocine binding to σ_1_ receptors. Taken together, these data suggest that dextromethorphan exerts some of its antidepressant actions through σ_1_ receptors.

## Introduction

Depression affects up to one fifth of the world population, stands as the second leading cause of disability worldwide, and imposes a substantial economic burden [1], [2]. In addition, the available pharmaceutical agents for treating depression are not effective in approximately a third of patients [3] and have a delayed clinical efficacy of several weeks to months [4]. Consequently, there is still a great need for faster acting and more effective treatments for depression.

Recently, a hypothesis was offered that dextromethorphan may have fast-acting antidepressant activity based on pharmacodynamic similarities to the N-methyl-D-aspartate (NMDA) antagonist ketamine [5], a drug repeatedly shown in human populations to display rapid antidepressant effects but whose use is severely limited by the need for intravenous administration and the presence of notable adverse effects (e.g., hallucinations and dissociations) [6], [7], [8]. Similar to ketamine, dextromethorphan binds to NMDA receptors and can modulate glutamatergic signaling [5]. Dextromethorphan also has higher affinity than ketamine for serotonin transporters (SERT) [9] and several other protein targets, including sigma-1 (σ_1_) receptors [5], [9] which have been proposed as therapeutic targets for antidepressant drugs [10]. Unlike ketamine, however, dextromethorphan has a high margin of safety; it has been used as a nonprescription antitussive over the past 40 years and thus may serve as a safer alternative to ketamine. In addition, it readily undergoes first-pass metabolism by cytochrome P450 (CYP) 2D6 to its major active metabolite dextrorphan [11]. Dextromethorphan in combination with quinidine, which raises the plasma concentration and bioavailability of dextromethorphan through the inhibition of CYP2D6 metabolism [12], is approved by the U.S. Food and Drug Administration (FDA) and European Medicines Agency (EMA) for the treatment of pseudobulbar affect and is thought to produce part of its therapeutic effects through σ_1_ receptors [13].

σ_1_ Receptors are highly conserved 223 amino acid proteins expressed on the mitochondrial-associated endoplasmic reticulum membrane (MAM) and can translocate between different cellular compartments in response to ligand binding [14]. In addition, σ_1_ receptors appear to operate primarily via protein-protein interactions to modulate the activity of various ion channels and signaling molecules, including inositol triphosphates, protein kinases, and calcium [14], [15].

Previous reports implicate σ_1_ receptors as protein targets for existing and novel antidepressant drugs [10]. Currently marketed antidepressant drugs, such as tricyclic antidepressants, monoamine oxidase inhibitors, selective serotonin reuptake inhibitors (SSRIs), and newer generations of antidepressant drugs, bind to these receptors [10]. Earlier studies also demonstrate that σ_1_ receptor agonists can modulate the activities of neurotransmitter systems, signaling pathways and brain regions implicated in the pathophysiology of depression [10] and that σ_1_ receptor knockout mice exhibit a depressive-like phenotype [16].

The potential clinical relevance of these observations is further supported by reports that σ_1_ receptor agonists produce antidepressant effects in experimental animals and humans [17], [18], [19], [20], [21], [22]. Notably, the σ_1_ receptor agonist igmesine hydrochloride proved to be as effective an antidepressant as the well-established SSRI fluoxetine in some clinical trials, though not in all cases [10], [22]. Compared to existing medications, σ_1_ receptor agonists may facilitate a more rapid onset of antidepressant efficacy [23]. Consistent with this, σ_1_ receptor agonists such as (+)-pentazocine and SA 4503 can enhance serotonergic neuronal firing in the dorsal raphe nucleus after only two days of treatment, compared to the two weeks of treatment that is typically required of conventional antidepressant drugs [24], [25].

In the studies herein, we test the hypothesis that dextromethorphan can exert antidepressant-like actions at least in part through σ_1_ receptors. This activity may convey additional therapeutic advantages over ketamine under clinically relevant conditions since compared to ketamine which has micromolar affinity for σ_1_ receptors [26], dextromethorphan exhibits nanomolar binding affinity for these receptors [9], [27], [28]. First, the ability of dextromethorphan to cause antidepressant-like effects was examined in the forced swim test. The forced swim test is the most validated behavioral assay for predicting antidepressant efficacy [3], [29], [30] and thus provides a rational format for the initial evaluation of the antidepressant potential of dextromethorphan. In addition, the effect of dextromethorphan on locomotor activity was measured to determine whether stimulant effects could account for its apparent antidepressant-like actions. The antidepressant drugs imipramine and fluoxetine were used as reference ligands for these behavioral tests. Second, to evaluate the potential involvement of σ_1_ receptors in the *in vivo* antidepressant-like actions of dextromethorphan, pharmacological antagonists targeting σ_1_ receptors were examined for their ability to prevent the antidepressant-like effects of dextromethorphan. Third, since dextromethorphan undergoes extensive first-pass metabolism by CYP2D6 to its major active metabolite dextrorphan [11], the CYP2D6 inhibitor quinidine was administered concomitantly with dextromethorphan to raise the plasma concentration and bioavailability of dextromethorphan [12] and determine whether the metabolism of dextromethorphan affects its antidepressant efficacy. Finally, to define the manner in which dextromethorphan binds to σ_1_ receptors (competitive and/or non-competitive), saturation binding studies were conducted.

## Materials and Methods

### Animals

Male, Swiss Webster mice (24–28 g; Harlan, Frederick, MD) were housed with food and water *ad libitum*, with a 12:12 h light–dark cycle. Animals were housed in groups of five for at least one week prior to initiation of experiments. All procedures were conducted in strict accordance with the recommendations in the Guide for the Care and Use of Laboratory Animals of the National Institutes of Health. The protocol was approved by the Institutional Animal Care and Use Committee at West Virginia University (Morgantown, WV), and all efforts were made to minimize suffering.

### Drugs and Chemicals

Dextromethorphan hydrobromide and quinidine sulfate were provided by Avanir Pharmaceuticals, Inc. (Aliso Viejo, CA; for the behavioral studies) or purchased from Sigma-Aldrich (St. Louis, MO; for the binding assays). Imipramine hydrochloride was purchased from Sigma-Aldrich (St. Louis, MO). Fluoxetine hydrochloride, BD1063 (1-[2-(3,4-dichlorophenyl)ethyl]-4-methylpiperazine dihydrochloride), and BD1047 (N-[2-(3,4-dichlorophenyl)ethyl]-N-methyl-2-(dimethylamino)ethylamine dihydrobromide) were obtained from Tocris (Ellisville, MO). [^3^H](+)-Pentazocine (34.8 Ci/mmol) was procured from Perkin Elmer (Hopkington, MA). All other chemicals and reagents were purchased from standard commercial suppliers (Sigma-Aldrich, St. Louis, MO).

### Drug Treatments

Mice (*N* = 5–15/group) received intraperitoneal (i.p.) injections with the following treatments: (1) Saline; (2) Imipramine (10–20 mg/kg); (3) Fluoxetine (10–30 mg/kg); (4) Dextromethorphan (1–30 mg/kg); (5) BD1063 (3–30 mg/kg); (6) BD1047 (10–30 mg/kg); (7) BD1063 (10 mg/kg) + Imipramine (20 mg/kg); (8) BD1063 (10 mg/kg) + Dextromethorphan (10–50 mg/kg); (9) BD1047 (10–20 mg/kg) + Dextromethorphan (30 mg/kg); (10) Quinidine (30 mg/kg) + Saline; and (11) Quinidine (30 mg/kg) + Dextromethorphan (3–30 mg/kg). Treatment with quinidine was administered concurrently with saline or dextromethorphan. Treatment with a σ_1_ receptor antagonist (BD1063 or BD1047) was administered 15 min prior to the second drug (imipramine or dextromethorphan).

### Locomotor Activity

Locomotor activity was measured utilizing an automated activity monitoring system (San Diego Instruments, San Diego, CA). Prior to locomotor activity measurements, animals were acclimated to the testing facility for at least 30 min and habituated to the testing chambers for an additional 30 min. Each testing chamber consisted of a Plexiglas housing and a 16×16 photobeam array to detect lateral (ambulatory and fine) movements, with a separate 16 photobeam array to detect rearing activity. Subsequent to the acclimation period, animals were treated and placed back in their respective chambers. Ambulatory, fine and rearing movements were quantified and summated as a measure of total locomotor activity for the next 30 min.

### Forced Swim Test

Immediately after the locomotor measurements, animals were placed in individual cylinders of water (10 cm deep) for a total of 6 min for the forced swim test. The initial 2 min was an acclimation period and not scored. During the remaining 4 min, immobility time was quantified using ANY-Maze Version 4.63 video tracking software (Stoelting Co., Wood Dale, IL). Immobility was defined as no activity other than that required to maintain the animal's head above the surface of the water. ANY-Maze software settings were as follows: accustomization period  = 120 s, test duration  = 240 s, minimum immobility time  = 2000 ms (2 s), and immobility sensitivity  = 75%.

### Saturation Binding Assays

To determine K_d_ and B_max_ by saturation binding, assays were performed in the absence (control) and presence of dextromethorphan (400 nM) using methods previously published in detail [31]. The concentration of dextromethorphan used in these assays was based on the reported K_i_ of the drug for σ_1_ receptors [27]. Briefly, 15 concentrations of [^3^H](+)-pentazocine (0.1–100 nM) were used to label σ_1_ receptors in P_2_ rat brain homogenates (400–500 µg/sample). Non-specific binding was determined in the presence of 10 µM haloperidol. Incubations occurred for 120 min at 25°C and membranes were washed 2–3 times using ice cold 10 mM Tris HCl, pH 8.0.

### Data Analysis

Data from all experiments were analyzed using GraphPad Prism 5.0 (San Diego, CA). The behavioral data were analyzed by one-way analysis of variance (ANOVA) followed when applicable by *post-hoc* Dunnett's or Tukey's multiple comparison tests. The correlation between locomotor activity and immobility time was analyzed by Pearson's r correlation test. The K_d_ and B_max_ were determined using nonlinear regression and analyzed by unpaired t-tests. For *in vivo* data, outliers (data points that were at least two standard deviations away from the mean) were excluded from analyses. Data are represented as mean ± S.E.M. *P*<0.05 was considered statistically significant for all data analyzed.

## Results

### Conventional Antidepressants Dose Response

**Figure 1:**
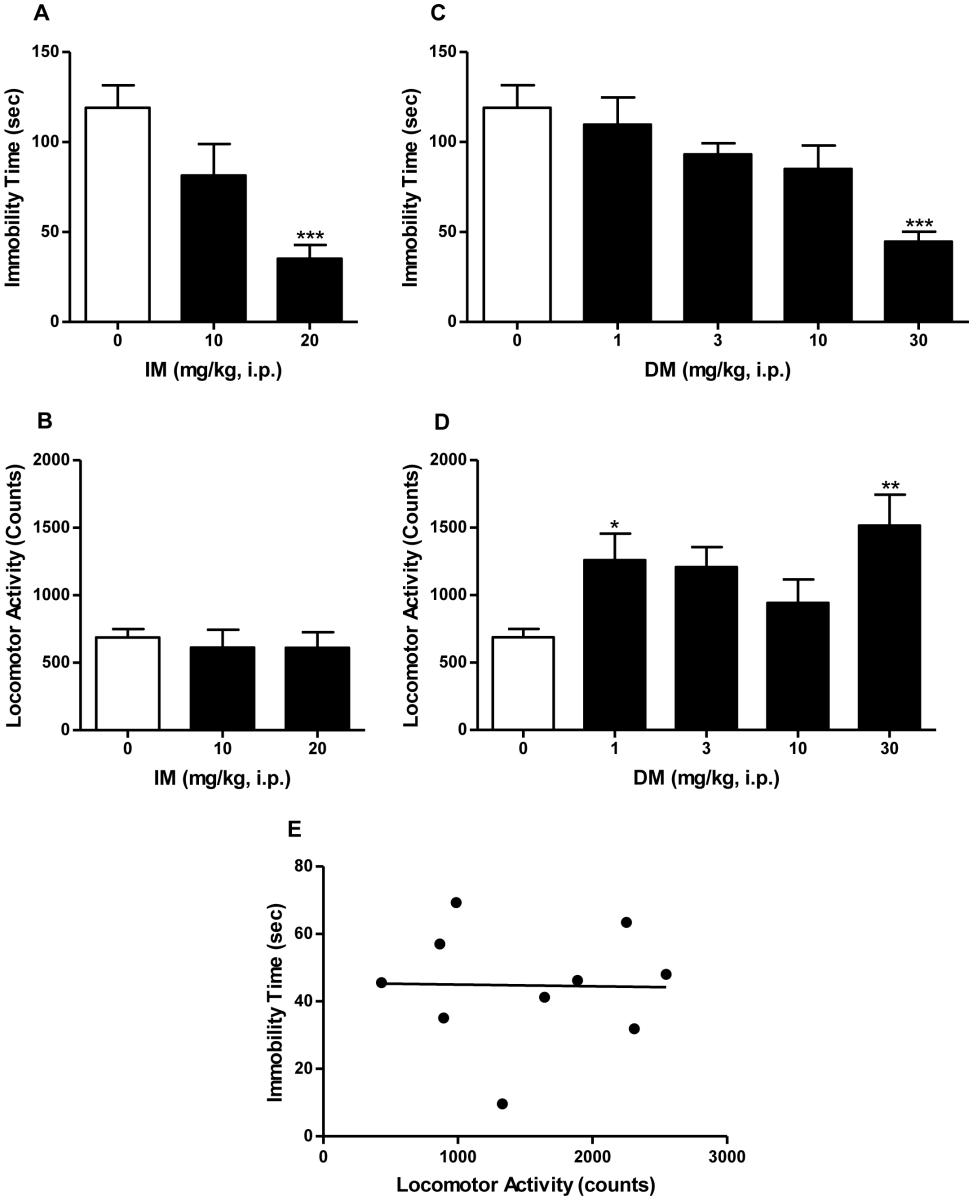
Antidepressant-like effects of imipramine and dextromethorphan in the forced swim test in mice. Imipramine (0–20 mg/kg, i.p.) significantly decreased immobility time (A), but had no significant effects on locomotor activity (B). Dextromethorphan (0–30 mg/kg, i.p.) significantly decreased immobility time (C), and significantly increased locomotor activity (D). However, there was no correlation between dextromethorphan (30 mg/kg)-induced locomotor stimulatory effects and decreased immobility times (E). Data shown are expressed as mean ± S.E.M. *P<0.05, **P<0.01, ***P<0.001, compared with the saline-treated group; one-way ANOVA followed by *post-hoc* Dunnett's tests. Pearson's r correlation test for correlation analysis. IM, imipramine. DM, dextromethorphan.

The tricyclic antidepressant imipramine served as a positive control and significantly altered immobility time in the forced swim test (Figure 1A; *F* [2], [32] = 10.24, *P*<0.05). *Post-hoc* Dunnett's test showed that imipramine at 20 mg/kg significantly decreased immobility time when compared to saline (*q* = 4.52, *P*<0.001). Imipramine did not alter locomotor activity, even at doses that produced antidepressant-like effects (Figure 1B; *F* [2], [30] = 0.23, n.s.).

Consistent with earlier reports that the forced swim test does not reliably detect the effects of SSRIs [37], the SSRI fluoxetine did not significantly reduce immobility time in the forced swim test under the conditions used by our laboratory (*F* [2], [32] = 1.97, n.s.). Fluoxetine also did not alter locomotor activity (*F* [2], [30] = 2.36, n.s.).

### Dextromethorphan Dose Response

Dextromethorphan significantly reduced immobility time (Figure 1C; *F*[4,50] = 6.16, *P*<0.001), with a *post-hoc* Dunnett's test revealing that 30 mg/kg differed significantly from saline (*q* = 4.70, *P*<0.05). Dextromethorphan also produced significant effects on locomotor activity (Figure 1D; *F* [4], [48] = 4.27, *P*<0.05), with *post-hoc* Dunnett's tests confirming that the following doses of dextromethorphan elicited effects that differed significantly from the saline control: 1 mg/kg (*q* = 2.63, *P*<0.05) and 30 mg/kg (*q* = 3.81, *P*<0.01). Since dextromethorphan showed significant stimulant effects, a correlation analysis between locomotor activity and immobility time was carried out to determine whether stimulant effects could account for its apparent antidepressant-like actions. The Pearson's r correlation test revealed that there was no correlation between the dextromethorphan-induced increase in locomotor activity and decrease in immobility time (Figure 1E; *r* = −0.02, n.s.)

### Sigma-1 Receptor Antagonists Dose Response

When tested alone, the σ_1_ receptor antagonist BD1063 displayed a significant effect on immobility time (*F* [3], [36] = 4.11, *P*<0.05). *Post-hoc* Dunnett's tests showed that BD1063 significantly reduced immobility time at 30 mg/kg (*q* = 3.41, *P*<0.01), but not at 10 mg/kg (*q* = 1.82, n.s.). Consequently, all antagonist testing in the forced swim test was performed with the 10 mg/kg dose of BD1063 which did not have effects on its own. Alone, BD1063 also produced significant changes in locomotor activity (*F* [3], [34] = 5.81, *P*<0.05). *Post-hoc* Dunnett's tests revealed that only the low 3 mg/kg, i.p. dose caused a significant increase in activity for BD1063 (*q* = 2.73, *P*<0.05), thereby indicating that the alterations in locomotor behavior did not account for the changes observed in the forced swim tests.

The second σ_1_ receptor antagonist BD1047 alone displayed no significant effects in the forced swim test (*F* [3], [36] = 2.55, n.s.). In the locomotor studies, BD1047 produced an effect that significantly differed from saline injections (*F* [3], [34] = 6.99, *P*<0.001). *Post-hoc* Dunnett's tests revealed that only the low 3 mg/kg, i.p. dose caused a significant increase in activity for BD1047 (*q* = 3.70, *P*<0.01).

### Effects of Sigma Receptor Antagonists in the Presence of Dextromethorphan or Imipramine

**Figure 2:**
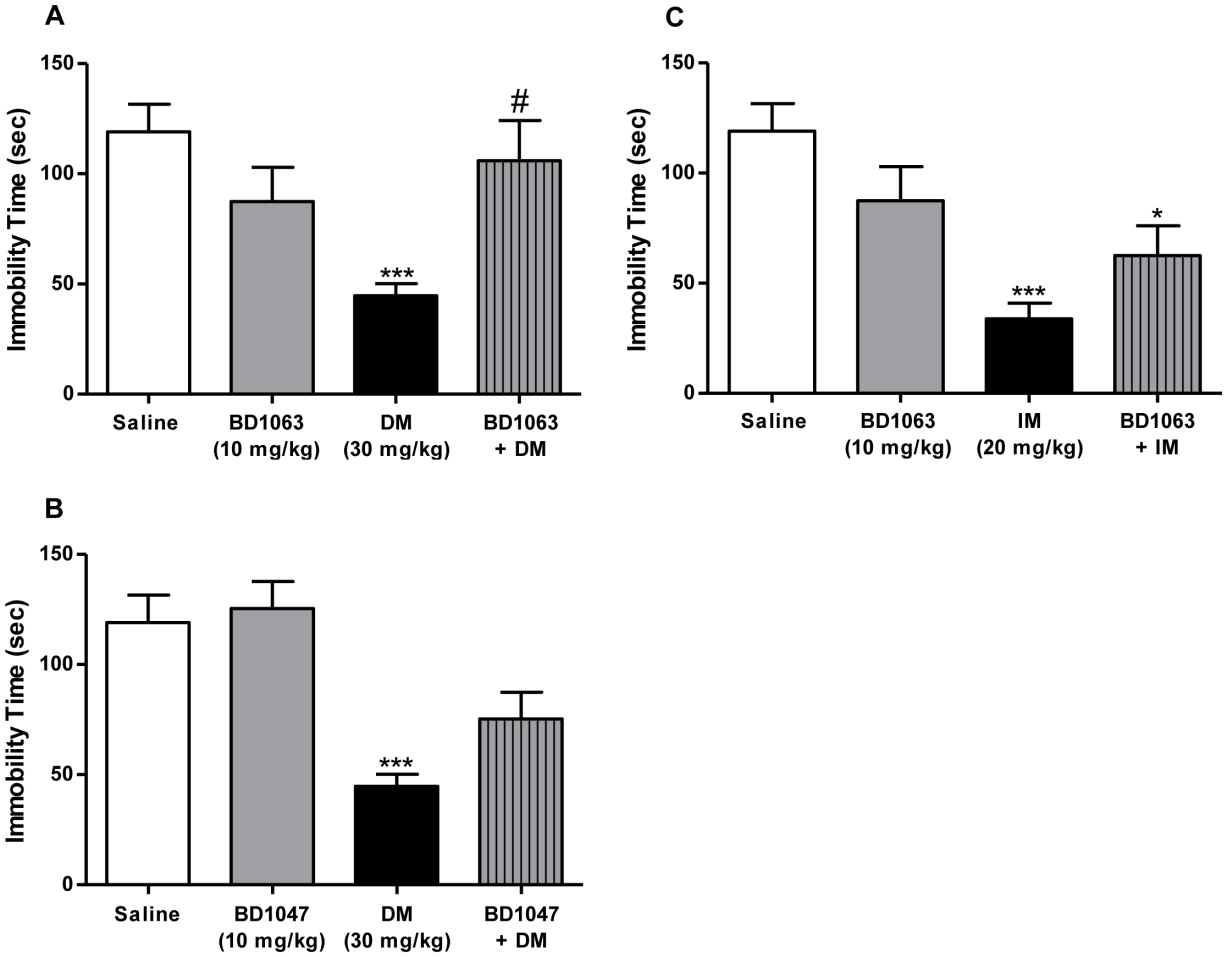
Attenuation of the antidepressant-like effects of dextromethorphan, but not imipramine, by σ_1_ receptor antagonism. Pretreatment with the σ_1_ receptor antagonist BD1063 (10 mg/kg, i.p.) prevented the dextromethorphan (30 mg/kg, i.p.)-induced decrease in immobility time (A). BD1047 (10 mg/kg, i.p.) pretreatment also produced a noticeable, albeit not statistically significant, trend toward the prevention of the decreased immobility time induced by dextromethorphan (B). In contrast, the antidepressant-like effect of imipramine (20 mg/kg, i.p.) in the forced swim test was not significantly prevented by BD1063 pretreatment (C). Data shown are expressed as mean ± S.E.M. *P<0.05, ***P<0.001, compared with the saline-treated group; #*P*<0.05, compared with the dextromethorphan-treated group; one-way ANOVA followed by *post-hoc* Tukey's tests. IM, imipramine. DM, dextromethorphan.

Pretreatment of mice with behaviorally inactive (10 mg/kg) doses of BD1063 or BD1047, two well-established and selective σ_1_ receptor antagonists, attenuated the antidepressant-like effects of dextromethorphan, but not imipramine (Figure 2). Pretreatment with BD1063 (10 mg/kg) attenuated the antidepressant-like effects of dextromethorphan (30 mg/kg) (Figure 2A). ANOVA confirmed a significant difference between the various treatment groups in the antagonism study for dextromethorphan in the forced swim test (*F* [3], [41] = 5.59, *P*<0.01). *Post-hoc* Tukey's multiple comparison tests revealed that the dextromethorphan alone treatment group differed significantly from saline (*q* = 5.60, *P*<0.001), as well as the BD1063 + Dextromethorphan group (Figure 2A; *q* = 4.21, *P*<0.05). Pretreatment of mice with BD1063 (10 mg/kg) also significantly decreased the locomotor stimulatory effect of dextromethorphan (30 mg/kg), with an overall significant difference between treatment groups (*F* [3], [39] = 10.03, *P*<0.0001). Moreover, *post-hoc* Tukey's test confirmed that BD1063 treatment blocked the ability of dextromethorphan to increase locomotor activity (*q* = 6.62, *P*<0.001).

Pretreatment with the second σ_1_ preferring antagonist BD1047 showed a trend toward the attenuation of the antidepressant-like effects of dextromethorphan (30 mg/kg), with the results of 10 mg/kg pretreatment with BD1047 illustrated in Figure 2B. The overall ANOVA was significant for the BD1047 pretreatment study (*F*[5,64] = 5.65, *P*<0.0005). Pairwise comparisons using *post-hoc* Tukey's multiple comparison tests further confirmed that the BD1047 (10 or 20 mg/kg) + Dextromethorphan groups did not differ significantly from saline (*q* = 4.04 and 2.77, n.s.). However, the differences in the effects of dextromethorphan in the absence and presence of BD1047 (10 or 20 mg/kg) were not significant (*q* = 2.52 and 3.07, n.s.), reflecting partial attenuation of the effects. In the locomotor studies, BD1047 significantly decreased the effects of dextromethorphan (*F*[5,61] = 6.59, *P*<0.0001), with *post-hoc* Tukey's tests confirming the ability of BD1047 (10 mg/kg) to block the stimulant effect of dextromethorphan (30 mg/kg) (*q* = 5.41, *P*<0.05).

In contrast to the attenuation of dextromethorphan-induced effects in the forced swim test, pretreatment with BD1063 did not prevent, although it non-significantly reduced, the antidepressant-like effects of imipramine (20 mg/kg) (Figure 2C). *Post-hoc* Tukey's test confirmed that imipramine significantly reduced immobility time compared to saline (*q* = 6.74, *P*<0.001). *Post-hoc* comparisons of the BD1063 + Imipramine group showed that its effects did not differ significantly from imipramine alone (*q* = 2.00, n.s.), and there was a significant reduction in immobility time compared to saline (*q* = 4.55, *P*<0.05). Locomotor activity differed among the treatment groups (*F* [3], [39] = 5.49, *P*<0.01). Although imipramine and BD1063 at the doses tested did not differ from saline (*q* = 1.01 and 0.13, respectively, n.s.), the combination of BD1063 + Imipramine produced a significant decrease in locomotor activity (*q* = 5.17, *P*<0.01).

### Effects of Sigma Antagonist BD1063 on Dextromethorphan Dose Response

**Figure 3:**
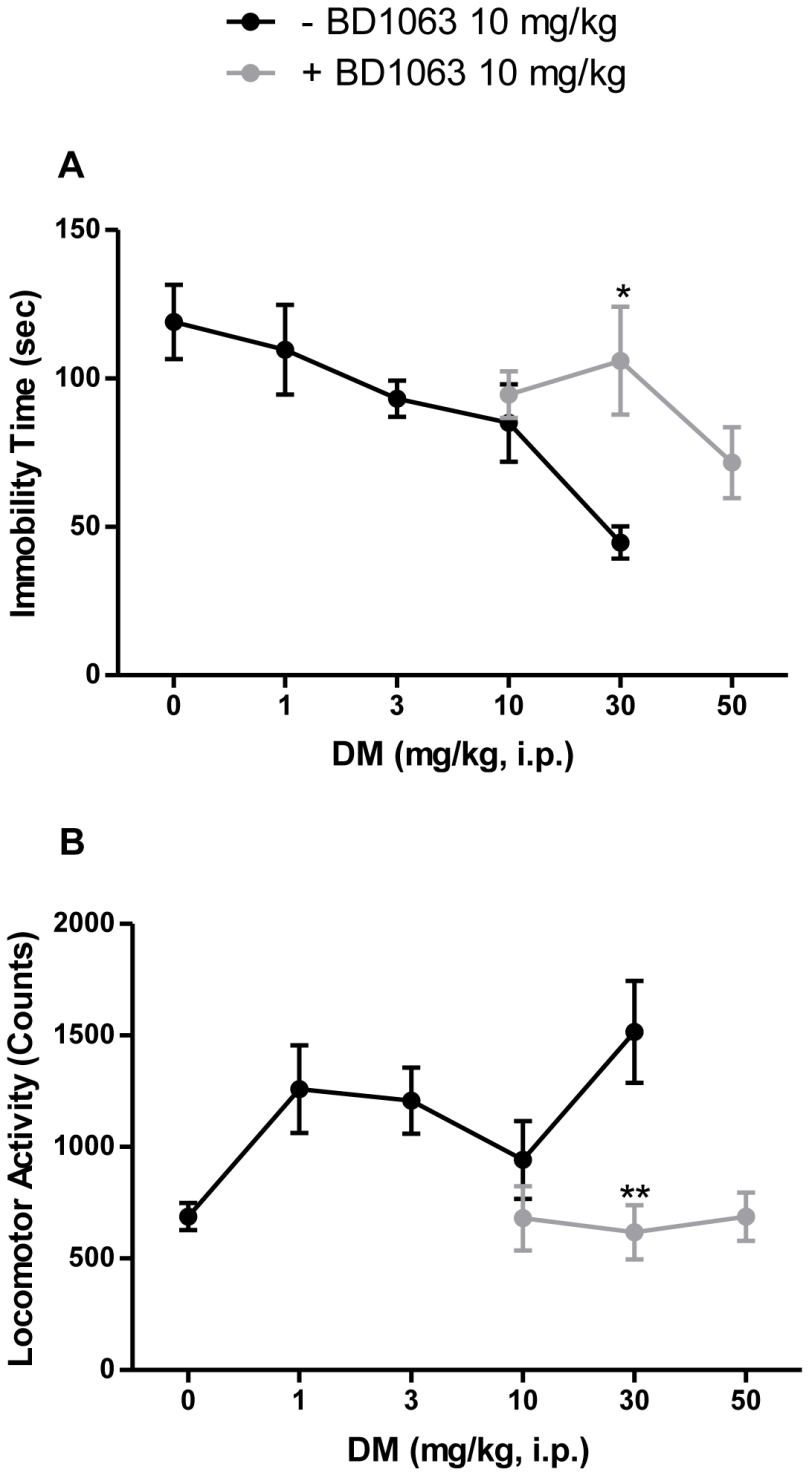
Competitive antagonism of the behavioral effects of dextromethorphan by BD1063. A single dose of BD1063 (10 mg/kg, i.p.) pretreatment shifted the dextromethorphan (0–50 mg/kg, i.p.) dose response curve to the right in the forced swim test (A), and blocked the dextromethorphan-induced stimulatory effect in the locomotor study (B). Data shown are expressed as mean ± S.E.M. *P<0.05, **P<0.01 compared with the dextromethorphan (30 mg/kg, i.p.)-treated group; one-way ANOVA followed by *post-hoc* Tukey's tests. DM, dextromethorphan.

When testing a single dose of BD1063 (10 mg/kg) against different doses of dextromethorphan, the presence of BD1063 elicited a shift to the right in the dextromethorphan dose response curve (Figure 3A). However, the animals exhibited abnormal behaviors, particularly vocalization, during the locomotor tests beginning at the 50 mg/kg dose of dextromethorphan in combination with BD1063. Higher doses of dextromethorphan were lethal, which limited the extent to which the dose response could be characterized. In locomotor studies, when testing the single dose of BD1063 (10 mg/kg) against increasing doses of dextromethorphan, BD1063 appeared to block the dextromethorphan-induced locomotor activity (Figure 3B). However, as mentioned above, the testing of higher doses of dextromethorphan was not carried out due to behavioral toxicity.

### Effects of CYP2D6 Inhibitor Quinidine on Dextromethorphan Dose Response

**Figure 4:**
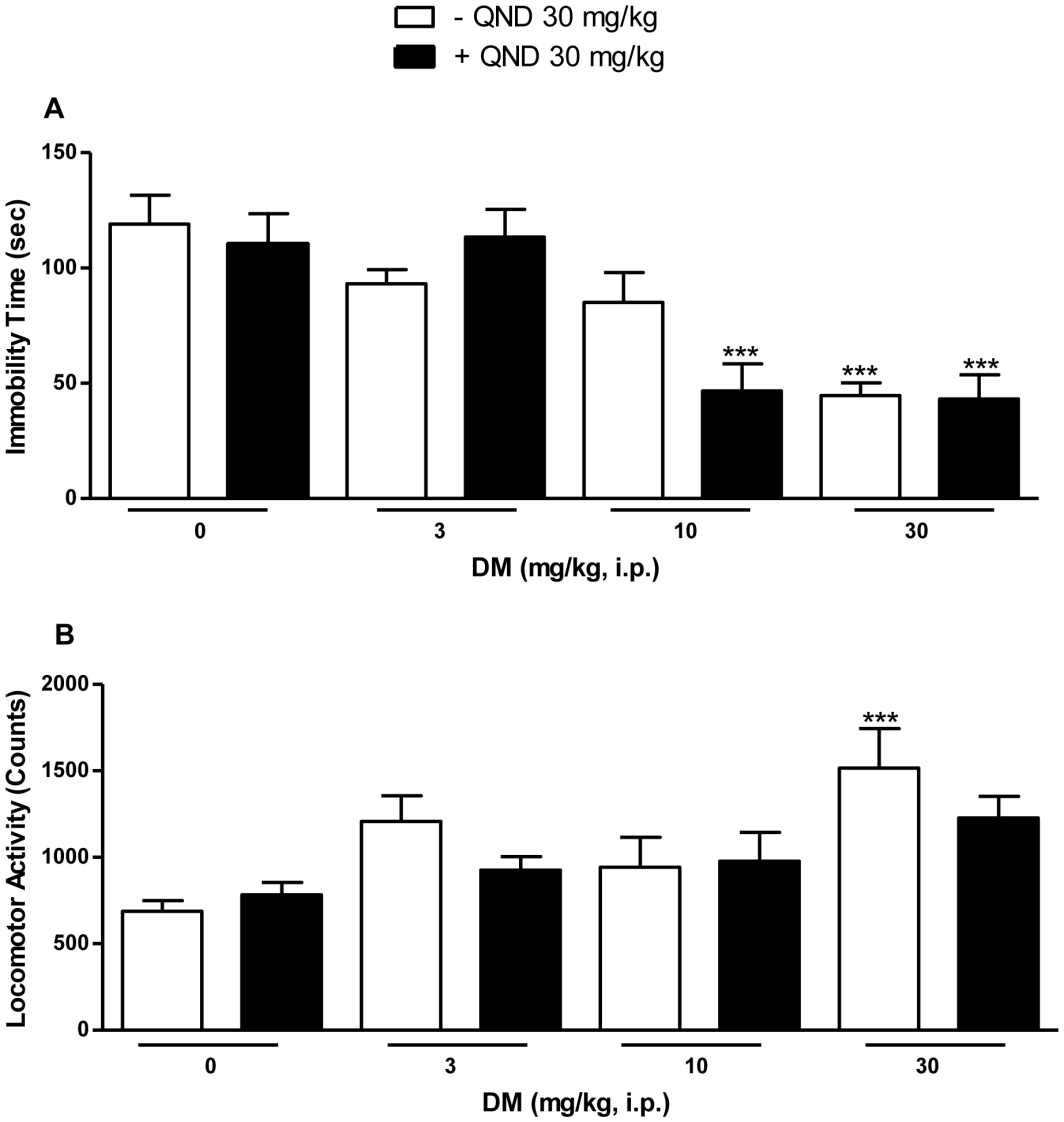
Potentiation of the antidepressant-like effects of dextromethorphan by quinidine. A single dose of the CYP2D6 inhibitor quinidine (30 mg/kg, i.p.) administered concomitantly with dextromethorphan (0–30 mg/kg, i.p.) significantly potentiated the decrease in immobility time for dextromethorphan at 10 mg/kg (A). In contrast, in the locomotor study, dextromethorphan in combination with quinidine had no stimulatory effects (B). Data shown are expressed as mean ± S.E.M. ***P<0.001, compared with the saline-treated group; one-way ANOVA followed by *post-hoc* Tukey's tests. QND, quinidine. DM, dextromethorphan.

Concurrent administration of quinidine (30 mg/kg), which blocks the CYP2D6 metabolism of dextromethorphan [12], potentiated the antidepressant-like effect of dextromethorphan (10 mg/kg) (Figure 4A). ANOVA confirmed a significant difference between the various treatment groups (*F*[7,77] = 8.45, *P*<0.0001). *Post-hoc* Tukey's multiple comparison tests revealed that the dextromethorphan (30 mg/kg) treatment group differed significantly from saline alone in the absence of quinidine (*q* = 6.96, *P*<0.001) and in combination with quinidine (*q* = 7.11, *P*<0.001). Importantly, while dextromethorphan at 10 mg/kg alone was not significantly different from saline (*q* = 3.19, n.s.), it produced a significant decrease in immobility time when combined with quinidine (*q* = 6.78, *P*<0.001). In locomotor studies, dextromethorphan in combination with quinidine had no stimulant effects (Figure 4B). The overall ANOVA was significant for the various treatment groups (*F*[7,73] = 3.97, *P*<0.001). Pairwise comparisons using *post-hoc* Tukey's multiple comparison tests further confirmed that the dextromethorphan (30 mg/kg) alone treatment group differed significantly from saline *(q* = 6.40, *P*<0.001). In combination with quinidine, however, dextromethorphan had no significant stimulant effects (*q* = 4.04, n.s.). When tested alone, quinidine had no significant effects in the forced swim test (*t* = 0.47, n.s.) nor did it have any effect on locomotor activity (*t* = 0.99, n.s.).

### Dextromethorphan Binding to Sigma-1 Receptors

The results of the σ_1_ receptor saturation binding assays demonstrated there was a significant reduction in K_d_ and B_max_ when the assays were performed in the presence of dextromethorphan, compared to when no dextromethorphan was added to the assay (Table 1). Unpaired t-tests revealed a significant decrease in both K_d_ (*t* = 3.87, *P*<0.05) and B_max_ (*t* = 4.29, *P*<0.05) with the inclusion of 400 nM dextromethorphan in the assays.

**Table 1:** Binding parameters for σ_1_ receptors in the absence and presence of dextromethorphan.

Assay condition	K_d_ (nM)	B_max_ (fmol/mg protein)
No additional compound	27.38±2.23	356±12
+ Dextromethorphan 400 nM	16.81±1.58^a^	290±9^a^

Saturation binding assays in brain homogenates for σ_1_ receptors were conducted using [^3^H](+)-pentazocine as the radioligand. The assays were performed in the absence or presence of dextromethorphan (400 nM). The K_d_ and B_max_ were determined using nonlinear regression. Dextromethorphan produced a significant decrease in K_d_ and B_max_. Data shown are expressed as mean ± S.E.M. ^a^ *P*<0.05, compared with [^3^H](+)-pentazocine alone; paired t-test.

## Discussion

This study is the first to show that dextromethorphan has antidepressant-like effects *in vivo*, in addition to implicating σ_1_ receptors as a mechanism contributing to its antidepressant actions. Moreover, concomitant administration of the CYP2D6 reversible inhibitor quinidine potentiated the effects of dextromethorphan in the forced swim test. This demonstrates that the antidepressant-like effects of dextromethorphan do not require conversion to the metabolite dextrorphan, and reveals dextromethorphan itself has antidepressant efficacy.

The antidepressant-like effects of dextromethorphan appear to involve σ_1_ receptors. In the current study, two well-established σ_1_ receptor antagonists (BD1063 and BD1047) reduced the antidepressant-like actions of dextromethorphan *in vivo*. They are thought to act in a competitive manner since in the presence of BD1063, the dose response curve for dextromethorphan was shifted to the right. An involvement of σ_1_ receptors in the antidepressant-like effects of dextromethorphan is consistent with earlier reports that selective σ_1_ receptor agonists can on their own reduce immobility time in the forced swim test [17], [20], [21], [32] and produce antidepressant-like effects in other animal models such as the tail suspension test and olfactory bulbectomy [18], [33]. Thus, additional studies involving these and other animal models used in depression research (e.g., sucrose preference test, novelty suppression) [3], [29], [30] will be needed in the future to further evaluate the antidepressant potential of dextromethorphan and the involvement of σ_1_ receptors.

The ability of dextromethorphan to elicit antidepressant-like actions through σ_1_ receptors suggests future studies to evaluate potential fast-acting therapeutic effects are also warranted. σ_1_ Receptor agonists can enhance serotonergic neuronal firing in the dorsal raphe nucleus after only two days vs. two weeks of treatment that is typically required of conventional antidepressant drugs [24], [25]. In addition, the fast-acting antidepressant drug ketamine has recently been shown to potentiate nerve growth factor (NGF)-induced neurite outgrowth through a σ_1_-dependent mechanism [26], supporting the emerging importance of σ_1_ receptors in modulating neuronal plasticity, which itself is a critical element for conveying both rapid and delayed antidepressant activity.

Earlier competition binding studies showed that dextromethorphan has significant affinity for σ_1_ receptors (138–652 nM) [13], [27], [28], [34], and thus further characterization of the interaction of dextromethorphan with σ_1_ receptors was undertaken in the current study. The saturation binding studies indicate that the interaction of dextromethorphan with σ_1_ receptors is complex, with both a change in B_max_ and K_d_ in the binding of [^3^H](+)-pentazocine in the presence of dextromethorphan. The reduction in the number of σ_1_ receptors (B_max_) with which [^3^H](+)-pentazocine binds suggests non-competitive interactions of dextromethorphan with σ_1_ receptors. However, there is also a decrease in K_d_ for [^3^H](+)-pentazocine binding in the presence of dextromethorphan, suggesting additional competitive interactions. Together, the data support the presence of at least two distinct sites or modes of interaction with which dextromethorphan binds to the σ_1_ receptor, one with which it has competitive interactions, and another with which it has non-competitive interactions. This interpretation would be consistent with other reports of multiple regions for ligand interactions on the σ_1_ receptor, some of which have functional ramifications for agonist vs. antagonist activity [35], [36], [37]. The affinity differences of dextromethorphan for its two putative binding sites appear to be similar (<100-fold difference) since competition binding assays of dextromethorphan at σ_1_ receptors are consistent with a one-site fit [27]. The antidepressant-like effects of dextromethorphan are thought to be mediated through the competitive binding site since i) there appears to be a rightward shift in its dose response curve in the forced swim test with no apparent change in maximal effect, and ii) (+)-pentazocine, the σ_1_ agonist used to label the receptor, has previously also been reported to produce similar antidepressant-like effects [10], [17].

In addition to interacting with σ_1_ receptors, dextromethorphan has been reported to alter monoamine reuptake, particularly serotonin and norepinephrine at Ki values of 23 and 240 nM, respectively [38], which have implications for antidepressant effects in humans. The significant affinity of dextromethorphan for SERT (40 nM) [9] would be expected to contribute to antidepressant efficacy in humans, although it would not account for potential fast-acting effects, nor reductions in immobility time herein. Under the experimental parameters used in the current study, the classical SSRI fluoxetine did not produce significant reductions in immobility time in the forced swim test. This is consistent with the reports of others that the forced swim test does not reliably detect the antidepressant potential of SSRIs [39]. Thus, this mechanism, which is a known contributor to antidepressant efficacy in humans, is unlikely to account for the pattern of antidepressant-like effects observed with dextromethorphan herein. In contrast to its high affinity for SERT, dextromethorphan binds much more weakly with NET (>1 µM) [9], but its reported ability to modulate norepinephrine reuptake [38] would be expected to contribute conventional antidepressant effects under clinical conditions.

Compared to the ability of BD1063 pretreatment to significantly block the antidepressant-like effects of dextromethorphan, it failed to attenuate that of imipramine, which has an overlapping binding profile with dextromethorphan: SERT (1.3–20 nM) [40], [41], [42], [43], and σ_1_ receptors (343 nM) [44]. This indicates that the σ_1_ interaction may have a larger role in producing the antidepressant-like effects of dextromethorphan than that of imipramine. This is consistent with the wider range of protein targets through which imipramine, but not dextromethorphan, interacts, which include: serotonin 5-HT_2_, muscarinic, and histamine H_1_ receptors [9], [45], [46], [47], [48], [49].

Finally, dextromethorphan elicits stimulant actions which were quantified herein as increases in locomotor activity. Two observations are of note with regard to these actions. First, the stimulant effects cannot account for the antidepressant-like actions of dextromethorphan. Second, quinidine enhances the antidepressant-like effects of dextromethorphan without producing an increase in locomotor activity. This suggests that addition of quinidine to dextromethorphan can be used clinically to optimize therapeutic antidepressant actions, without eliciting unwanted stimulant effects.

In conclusion, the data presented here show for the first time that dextromethorphan has antidepressant-like effects in an *in vivo* model and provide evidence that this effect occurs at least in part through a σ_1_ receptor dependent mechanism. This is also the first report of the manner in which dextromethorphan interacts at the σ_1_ receptor. Together with earlier studies and the potential of increasing dextromethorphan bioavailiabity by using the FDA- and EMA-approved dextromethorphan/quinidine formulation, these data suggest dextromethorphan should be further explored for translational potential as an antidepressant drug in clinical trials, as it may offer rapid-acting relief of depressive symptoms and the ability to resolve cases of treatment-resistant depression. In addition, further studies to understand the molecular and cellular mechanisms by which these effects occur are necessary and may yield important information about how various receptors, transporters and processes are involved in the ability of dextromethorphan to convey its antidepressant effects.
